# Vive la radiorésistance!: converging research in radiobiology and biogerontology to enhance human radioresistance for deep space exploration and colonization

**DOI:** 10.18632/oncotarget.24461

**Published:** 2018-02-12

**Authors:** Franco Cortese, Dmitry Klokov, Andreyan Osipov, Jakub Stefaniak, Alexey Moskalev, Jane Schastnaya, Charles Cantor, Alexander Aliper, Polina Mamoshina, Igor Ushakov, Alex Sapetsky, Quentin Vanhaelen, Irina Alchinova, Mikhail Karganov, Olga Kovalchuk, Ruth Wilkins, Andrey Shtemberg, Marjan Moreels, Sarah Baatout, Evgeny Izumchenko, João Pedro de Magalhães, Artem V. Artemov, Sylvain V. Costes, Afshin Beheshti, Xiao Wen Mao, Michael J. Pecaut, Dmitry Kaminskiy, Ivan V. Ozerov, Morten Scheibye-Knudsen, Alex Zhavoronkov

**Affiliations:** ^1^ Biogerontology Research Foundation, London, UK; ^2^ Department of Biomedical and Molecular Sciences, Queen's University School of Medicine, Queen's University, Kingston, Ontario, Canada; ^3^ Canadian Nuclear Laboratories, Chalk River, Ontario, Canada; ^4^ Department of Biochemistry, Microbiology and Immunology, University of Ottawa, Ottawa, Ontario, Canada; ^5^ Insilico Medicine, Inc., Emerging Technology Centers, Johns Hopkins University, Baltimore, MD, USA; ^6^ State Research Center - Burnasyan Federal Medical Biophysical Center of Federal Medical Biological Agency, Moscow, Russia; ^7^ Moscow Institute of Physics and Technology, Dolgoprudny, Russia; ^8^ Nuffield Department of Medicine, Target Discovery Institute, University of Oxford, Oxford, UK; ^9^ Laboratory of Molecular Radiobiology and Gerontology, Institute of Biology of Komi Science Center of Ural Branch of Russian Academy of Sciences, Syktyvkar, Russia; ^10^ Engelhardt Institute of Molecular Biology of Russian Academy of Sciences, Moscow, Russia; ^11^ Boston University, Department of Biomedical Engineering, Boston, MA, USA; ^12^ Laboratory of Bioinformatics, D. Rogachev Federal Medical Research Center of Pediatric Hematology, Oncology and Immunology, Moscow, Russia; ^13^ Computer Science Department, University of Oxford, Oxford, UK; ^14^ Laboratory of Physicochemical and Ecological Pathophysiology, Institute of General Pathology and Pathophysiology, Moscow, Russia; ^15^ Research Institute for Space Medicine, Federal Medical Biological Agency, Moscow, Russia; ^16^ Canada Cancer and Aging Research Laboratories, Ltd., Lethbridge, Alberta, Canada; ^17^ University of Lethbridge, Lethbridge, Alberta, Canada; ^18^ Environmental and Radiation and Health Sciences Directorate, Health Canada, Ottawa, Ontario, Canada; ^19^ Laboratory of Extreme Physiology, Institute of Medical and Biological Problems RAS, Moscow, Russia; ^20^ Radiobiology Unit, Interdisciplinary Biosciences, Institute for Environment, Health and Safety, Belgian Nuclear Research Centre, (SCK·CEN), Mol, Belgium; ^21^ Department of Molecular Biotechnology, Ghent University, Ghent, Belgium; ^22^ The Johns Hopkins University, School of Medicine, Department of Otolaryngology, Head and Neck Cancer Research, Baltimore, MD, USA; ^23^ Integrative Genomics of Ageing Group, Institute of Ageing and Chronic Disease, University of Liverpool, Liverpool, UK; ^24^ NASA Ames Research Center, Moffett Field, CA, USA; ^25^ Wyle Laboratories, Space Biosciences Division, NASA Ames Research Center, Mountain View, CA, USA; ^26^ Division of Hematology/Oncology, Molecular Oncology Research Institute, Tufts Medical Center, Boston, MA, USA; ^27^ Department of Basic Sciences, Division of Biomedical Engineering Sciences (BMES), Loma Linda University, Loma Linda, CA, USA; ^28^ Deep Knowledge Life Sciences, London, UK; ^29^ Center for Healthy Aging, University of Copenhagen, Copenhagen, Denmark

**Keywords:** radioresistance, space exploration, longevity, DNA damage, Mars mission

## Abstract

While many efforts have been made to pave the way toward human space colonization, little consideration has been given to the methods of protecting spacefarers against harsh cosmic and local radioactive environments and the high costs associated with protection from the deleterious physiological effects of exposure to high-Linear energy transfer (high-LET) radiation. Herein, we lay the foundations of a roadmap toward enhancing human radioresistance for the purposes of deep space colonization and exploration. We outline future research directions toward the goal of enhancing human radioresistance, including upregulation of endogenous repair and radioprotective mechanisms, possible leeways into gene therapy in order to enhance radioresistance via the translation of exogenous and engineered DNA repair and radioprotective mechanisms, the substitution of organic molecules with fortified isoforms, and methods of slowing metabolic activity while preserving cognitive function. We conclude by presenting the known associations between radioresistance and longevity, and articulating the position that enhancing human radioresistance is likely to extend the healthspan of human spacefarers as well.

## INTRODUCTION

Cosmic radiation and microgravity represent two major environmental contributors to human health risks and substantially limit the prospects of long spaceflights [1]. The necessity to protect the human body from the deleterious effects of cosmic radiation has been largely overlooked. For deep space exploration missions, including the mission to Mars, exposure to radiation represents one of several unacceptable risk categories [2, 3], since cumulative doses received by astronauts would greatly exceed the human dose limits established within the current NASA radiological protection system [4]. A 3% exposure-induced death risk limit, mostly from fatal radiation-induced cancer, is set according to this paradigm [3]. Implementation of additional protection systems including biotechnological concepts described here may help resolve this issue and make it possible to begin the era of deep space manned missions.

The main components of space radiation are solar particle events (SPE), geomagnetically trapped radiation and galactic cosmic radiation (GCR) [5]. The contribution of the first two to the total dose absorbed by astronauts would obviously be negligible on long-duration missions away from Earth and the Sun. Consequently, GCR consisting mainly of highly-energetic particles (reviewed below) would be the primary type of radiation encountered by humans under this scenario ([2, 3, 6]). It has been estimated that a return trip to Mars could subject astronauts to radiation doses of 660 mSv [7]. Although great uncertainties exist with respect to health (cancer) risk estimates from exposure to cosmic radiation [7, 8], this dose alone represents more than half of the total NASA astronaut career limit of 800-1200 mSv [9]. Obviously, longer missions would not be acceptable for human in terms of cancer risk under current radioprotection guidelines. Due to very high energies of charged particles of GCR they easily penetrate passive shielding materials. Although active shielding technologies are also currently being explored, there has been no substantial progress in significantly reducing fluxes of GCR down to levels suitable for long-duration human space missions [10, 11]. It is therefore important to explore various prospects of improving human radioresistance using recent advances in biotechnology. These would include the possibility of genetic modifications to humans utilizing breakthrough technologies in gene editing in combination with the current knowledge of molecular pathways counteracting radiation-induced DNA damage, as well as other possible therapies, such as regenerative medicine, low-dose radioadaptation, the use of deuterated organic compounds, hypostasis (considerable slowdown of all the vital processes in the body) or a combination thereof. Finally, the primary focus of this concept review is the radioprotection against DNA damage driven health risks, mainly cancer. Some of the ideas in this review could potentially be utilized for alleviating other detrimental effects of long space travel, such as muscle and bone deterioration.

In principle, ionizing radiation interacts along charged particle tracks with biological molecules such as DNA. The process is largely stochastic, and can damage DNA via direct interactions (e.g. ionization and excitation) or via indirect interactions such as through the production of reactive oxygen species (ROS) as a result of radiolysis of water molecules [12].

Radioresistance denotes the capacity for organisms to protect against, repair and remove molecular, cellular and tissue damage caused by ionizing radiation. It is a quality that varies greatly in terms of effectiveness between different organisms. For instance, it is well-known that certain organisms are remarkably resistant to the damaging effects of radiation. The bacterium *Deinococcus radiodurans*, for instance, possess error-free DNA repair mechanisms and can withstand doses as high as 7 kGy [13]. Similarly, tardigrades can withstand doses as high as 5 kGy, though doses exceeding 1 kGy render them sterile. Initially, the remarkable radioresistance of tardigrades was thought to be the result of their anhydrobiotic (i.e. dehydrated) state reducing the effective concentration of hydrolyzable water. However, subsequent studies have found that hydrated tardigrades were more radioresistant to both gamma-rays and heavy ions than anhydrobiotic specimens (the median lethal dose being 5 kGy for gamma-rays and 6.2 kGy for heavy ions in hydrated animals compared to 4.4 kGy for gamma-rays and 5.2 kGy for heavy ions an anhydrobiotic specimens) [14]. By comparison, the human median lethal dose is around 0.004 kGy.

Currently, the European Space Agency ESA is conducting intensive research on the possibility of the remote space missions. Given that the flight will take place mainly under the control of automatic systems, where the participation of the astronauts is almost not necessary, the space crew will be virtually imprisoned for many months without having any work to do. Such situations may be dangerous, especially for the astronauts themselves. Therefore, the ESA believes that it would be wiser to immerse people into hibernation. Currently ESA has started the project “Aurora”, where the option of crew hibernation is considered. The project team intends to engage the biological mechanisms that will allow the crew to sleep and thereby reduce the metabolism of the organism to the absolute minimum [15].

It is worth emphasizing that the idea of possible hibernation during the long space missions was also explored in the USSR in 1969, but, unfortunately, after the death of the father of Soviet cosmic program Sergei Korolev the Soviet manned “Mars project” was closed, and all the work related to its implementation was terminated. The results of these studies included data on the hyperresistance to various damaging factors including lethal doses of ionizing radiation, long-term lethal overloads and hypobaric hypoxia in mice [16].

## SPACE RADIATION ENVIRONMENT

Beyond the reach of the Earth's magnetosphere, cosmic rays refer to a heterogenous pool of ionizing radiation with a diverse range of energies and charges. They can be subdivided into two main types, including galactic cosmic rays (GCRs) consisting of a constant flux of high energy particles originating in cataclysmic astronomical events outside the solar system, and solar energetic particles produced by the Sun ([17]). GCRs mainly consist of approximately 85% high-energy protons, 12% alpha-particles, and 2% electrons. Importantly, there is a small (1%), but biologically relevant presence of high charge (Z) and energy (E) (HZE) nuclei [18]. Due to their high linear energy transfer (LET), this HZE component of GCRs represents a significant danger, and they are predicted to account for most of the biological consequences of cosmic radiation exposure [19, 20]. It has long been recognized that HZE ions have a higher relative biological effectiveness (RBE) compared to gamma-rays and must pose a significant cancer risk to humans in space [21].

In addition to GCRs, astronauts in space are exposed to solar energetic particles (SPEs), emitted by the Sun produced either via solar-flares or coronal mass ejections. SPEs are unpredictable and are largely comprised of low to medium energy protons.

**Figure 1:**
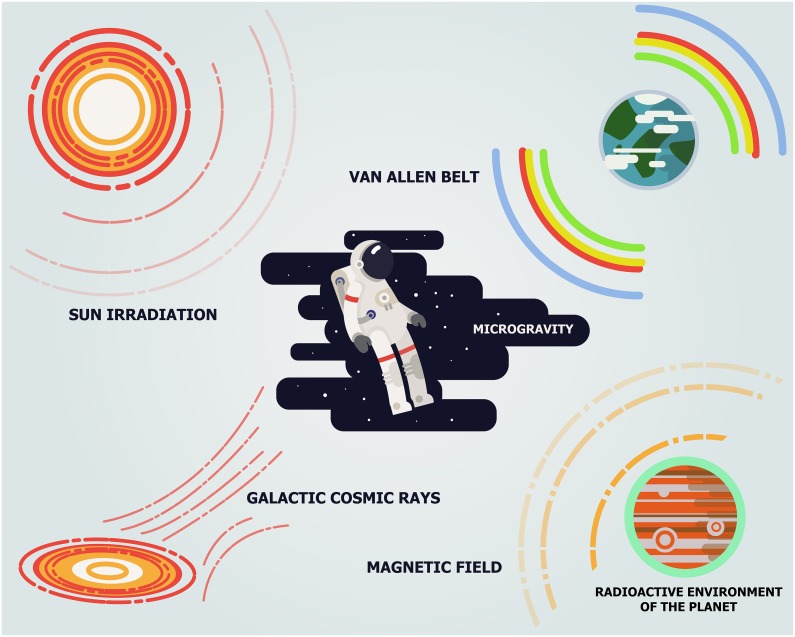
Major sources of space radiation The space radiation comes from three major sources including galactic cosmic rays, sun radiation and Van Allen radiation belts of the Earth.

Interaction of this primary cosmic radiation with the spacecraft shielding can create secondary radiation, mainly represented by neutrons, electrons, mesons and gamma-rays. This secondary radiation is thought to contribute to the overall radiation exposure as well [17, 18, 21, 22]. Recently, using NASA's models of risks and uncertainties, predictions were made for cancer and circulatory diseases related to space exploration missions to Mars near the solar minimum [2, 3]. It was estimated that radiation induced mortality and morbidity could exceed 5% and 10% with upper 95% confidence interval near 10% and 20% respectively for a Mars mission (Figure 1). Most recent estimates of cancer risks associated with Mars mission carried out using non-targeted effects model, thought to be more accurate at low-dose exposures, resulted in higher than previously assumed risks [23].

## MOLECULAR AND CELLULAR EFFECTS OF COSMIC RAYS AND MECHANISMS OF PROTECTION

Ionizing radiation's ability to induce genetic mutations had been established [24] well before the elucidation of the DNA double helix structure by Watson and Crick, or even before the Hershey-Chase experiment, which demonstrated that DNA is indeed the genetic material. The effects of ionizing radiation on DNA tend to be more serious than other biological macromolecules, with delayed systemic health effects being the outcome of DNA and chromosomal damage [25, 26].

The majority of cellular DNA lesions caused by ionizing radiation differ significantly from those caused by endogenous sources in their physical and chemical properties [27]. The most important features of radiation-induced DNA lesions are their complexity and clustering [28]. The radiation-induced DNA double-strand breaks (DSBs) tend to be the most crucial for the cell fate and can thus be considered to be a basic trigger for the cellular response and adaptation to the radiation exposure [29, 30]. Because eukaryotic cells primarily repair DSBs via the method of non-homologous end joining or allelic recombination (see below), there exists an unfortunate tendency to lose genetic information by means of loss of heterozygosity and deletions, as well as genetic rearrangements [31]. This can lead to serious cytogenetic lesions and cell death, or transformation and oncogenicity via the inactivation of tumor suppressor genes or the activation of certain oncogenes [32–34].

**Figure 2:**
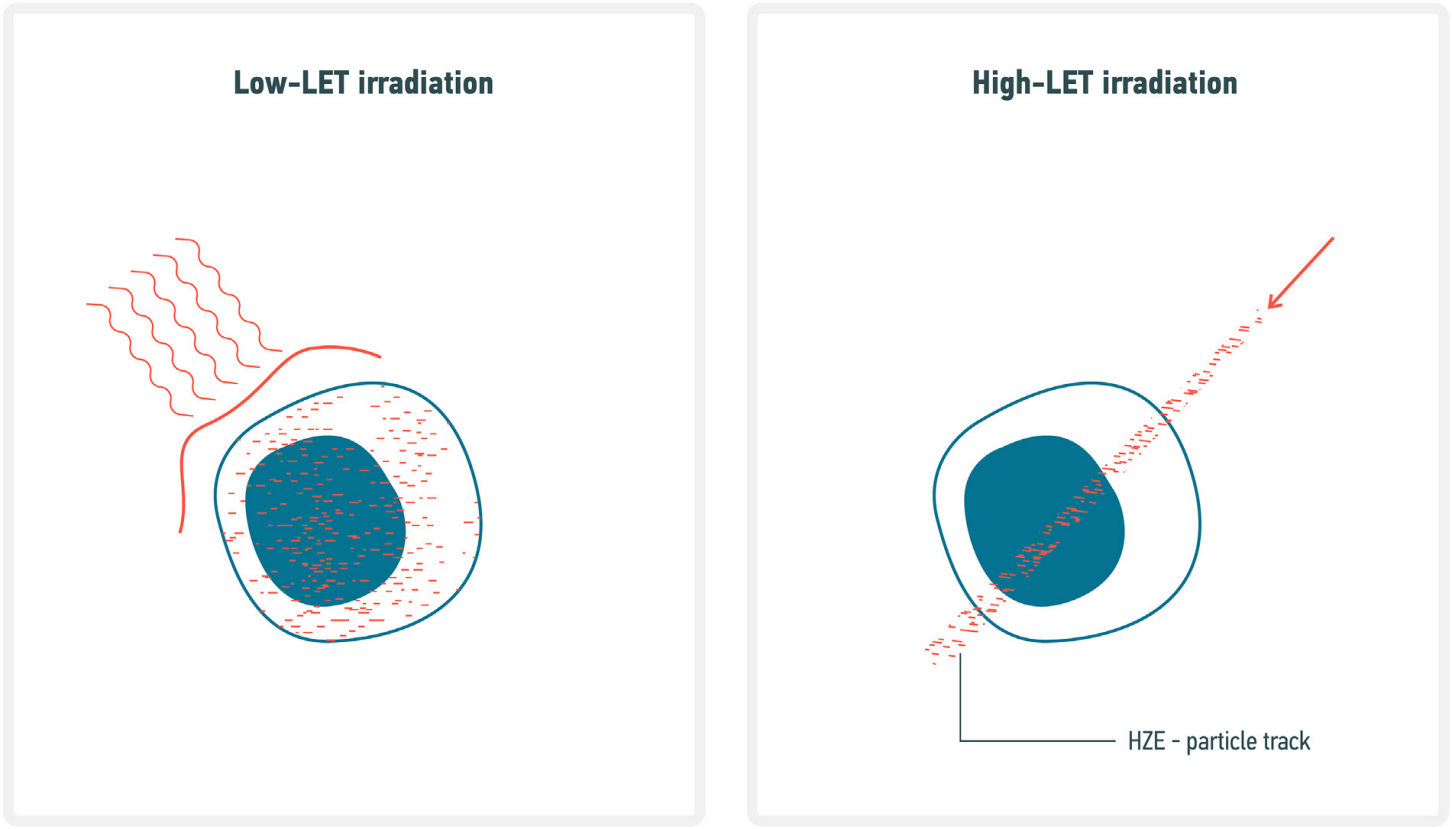
Comparative diagram on DNA damage induced by Low- and High-LET radiation HZE particles, also called “densely ionizing radiation” typically deposit a large amount of their energy along linear tracks referred to as cores, while the remaining energy is deposited radially and uniformly by secondary electrons (i.e. Delta-rays). In contrast, low-LET deposit their energy uniformly and are often referred as “sparsely ionizing radiation”.

LET denotes the amount of energy released by a particle or a photon over the length of its trajectory (typically in a unit of energy per unit length, i.e. keV/μm). As discussed above, Galactic Cosmic Rays are comprised of high charge and energy particles (referred to as HZE). The considerable speed of these particles makes them essentially electron-free (in other words they carry a high positive charge) and consequently, due to their Coulombic interactions with matter, they tend to decelerate on a linear track. In contrast to X rays, gamma rays, beta-particles and high energy neutrons, HZE ions have very high LET levels. HZE particles, also called “densely ionizing radiation” typically deposit a large amount of their energy along linear tracks referred to as cores, while the remaining energy is deposited radially and uniformly by secondary electrons known as Delta-rays. In contrast, low-LET particles deposit their energy uniformly and are often referred to as “sparsely ionizing radiation”. We have previously illustrated the difference of microdosimetry profiles between low and high-LET [35], by using the example of one 1 GeV/u 56Fe particle traversing an hypothetical square cell. In this example, with the size of the cell picked so that one Fe track traversal leads to an average dose of 1 Gy in the cell, the local dose within 0.5 μm radially from the track is 26 Gy whereas the rest of the cell is only exposed to a uniform dose of 0.17 Gy deposited by Delta-rays (Figure 2).

The most problematic type of DNA lesions induced by ionizing radiation are DNA double strand breaks (DSBs). Both low- and high-LET induce DSBs, but the complexity of damage varies with LET. We previously reviewed this topic in detail [36]. Briefly, if we accept the convention that a “simple DSB” is made of one break on each strand within a 10 base-pair region and “complex DSB” have at least one additional break between these two strand breaks, then roughly 80% of high-LET induced DSB are complex against only 20% for low-LET [37, 38]. The higher carcinogenic effect for high-LET [9, 39] has been explained primarily via the larger amount of complex break which hypothetically lead to more unrepairable lesions.

## RADIATION DNA DAMAGE AND MECHANISMS OF REPAIR

All eukaryotic organisms have evolved against a backdrop of constant exposure to endogenous and exogenous mutagens, and as such have developed robust cellular mechanisms for DNA repair and protection against DNA damage. The mechanisms of cellular protection against DNA damage and mutation and of DNA repair can be categorized as immediate and adaptive defense mechanisms.

Immediate defense mechanisms include:

- The production of antioxidants and reactive oxygen species (ROS) scavengers that neutralize ROS produced by oxygen metabolism, ionizing radiation and UV radiation [40–43].
- DNA repair [44–47], including direct reversal repair, base excision repair (BER) [48–51], nucleotide excision repair (NER) [52–54] and mismatch repair [44–47] for single strand modifications, and non-homologous end-joining (NHEJ), microhomology-mediated end-joining (MMEJ), homologous recombination for double strand breaks (DSBs), and interstrand crosslink repair for modifications affecting both strands [55–58];
- Elimination of damaged cells via apoptosis [59–64], and
- Proliferative arrest and replicative senescence [55–58, 65, 66]

Substantial experimental evidence suggests that low-dose radiation may trigger a variety of protective responses within cells, tissues and organisms that serve to protect them from both exogenous (e.g high doses of radiation) [67] and endogenous (e.g. age-related accumulation of DNA damage) [68] genomic instabilities [69–71]. Importantly, these responses, collectively termed radioadaptive responses or radiation hormesis, may protect against spontaneous or induced cancer [72]. Since radiation-induced tumorigenesis is a major risk concomitant with long-distance space travel, radiation hormesis has posed serious questions on the adequacy and scientific justification of the international radiation protection standards [70]. They dictate that any, however small, exposure to ionizing radiation leads to a proportional increase in cancer risk, thus postulating that there are no safe levels of radiation exposure. This system is based on the Linear-No-Threshold model that extrapolates human atomic bomb survival data obtained for high-to-low doses [73]. It appears that low-dose radiation-induced protective mechanisms are not specific to a certain molecular or cellular pathway and cover a broad range of cell-autonomous (DNA repair, anti-oxidant, pro-survival gene activation) and non-cell-autonomous mechanisms (immune system activation, bystander effects, etc.).

In relation to space, it is important to understand how radioadaptive responses can be elicited upon exposure to high LET radiation types. Early radioadaptive response studies failed to demonstrate beneficial biological responses upon exposure to low doses of high LET radiation [74]. However, more recently such a possibility has been demonstrated using human cell lines. Not only low-dose X-ray exposure was shown to trigger better DNA repair and cause lower mutation frequencies in response to high doses of high-LET ion irradiation [75], but also low doses of high-LET irradiation were reported to produce radiation adaptive responses upon high doses of the same high-LET irradiation [76]. Rithidech and Scott [77] proposed that the protective mechanisms could be triggered in cells by a gamma-component that is produced within a body upon exposure to neutrons [78]. Additionally, it has been shown that low doses of low LET radiation may render resistance to genotoxicity and teratogenesis induced by high doses of HZE particles [75, 79]. Intriguingly, priming normal human cells with low doses of energetic protons resulted in lower chromosomal damage induced by subsequent exposure to iron ions [80]. All of these scenarios are relevant to radiation exposures experienced by human astronauts onboard of a spacecraft, making it thus conceivable that cells and tissues may be made resistant to detrimental effects of GSR by priming or ongoing exposures to low LET radiation.

Genome instability resulting from DNA damage and mutation in both nuclear DNA and mitochondrial DNA caused by replication errors and exposure to endogenous and exogenous mutagens has long been implicated as one of the main causes of aging [81–86]. All of the above strategies for enhancing radioresistance in humans, from the expression and overexpression of exogenous and endogenous DNA repair genes, antioxidants and ROS scavengers, to the expression of exogenous radioprotective genes (e.g. Dsup), would also ancillarily serve as a means of attenuating DNA damage and mutation caused by exposure to the many endogenous and exogenous mutagens, foremost among these being endogenous ROS produced via normative cellular respiration, that are implicated in eukaryotic aging. As such, the above strategies for enhancing radioresistance in humans would also constitute a promising geroprotective strategy and a means of attenuating aging and promoting longevity and extension of both lifespan and healthspan in humans as well.

In addition to the direct effect of radiation on the target cells, a so-called bystander effect has been described where nearby, but unirradiated, cells activate their DNA damage response [87, 88]. These non-targeted effects are underlain by various mechanisms, for instance, at a systemic level via the microenvironment and TGF-beta signaling [89], via extracellular vesicles (EV), natural nanoparticles secreted by different cells and carrying protein markers, peptides, mRNA, microRNA (miRNA), non-coding RNA (ncRNA), DNA, and retrotransposon elements involved in cell-cell communication. Recently miRNAs have been shown to play an important role in radiation induced bystander effects [90]. Not only can miRNAs be contained in EV, but also can be free floating throughout the circulation. One study has shown that these circulating miRNAs contribute to non-targeted effects by traveling between irradiated and nonirradiated cells and increase the cells radioresistance [91].

In some studies, specific EV are secreted after irradiation, in particular, after radiation therapy. Experiments on cell cultures have demonstrated that EV and probably mRNA present in these EV mediate the development of the bystander effect [92–94]. In addition, a direct correlation between the amount of EV and irradiation dose has been demonstrated [95]. In experiments performed by Jella KK et al., exosomes (particles sized 30-100 nm) and microvesicles (>100 nm) were isolated after cell exposure to radiation in different doses and their concentration depended on the irradiation dose [93]. *In vitro* experiments have shown that radiation-induced EV are easily absorbed by cells during co-culturing and due to some modifications in their molecular composition promote cell migration by enhancing activation of TrkA and FAK signaling.

At the organism level, EV act as long-distance transport modules capable of crossing the blood-brain barrier [96]. EV also play an important role in the development of tumor process. Specific EV were isolated for human breast epithelial cancer, prostatic cancer, glioblastoma, pancreatic cancer, melanoma, and stomach cancer [92, 97–100].

When evaluating the effect of space flight factors, quantitative and qualitative characteristics of microparticles produced by different cells under normal and pathological conditions should be taken into account, as they considerably influence the development of genetic instability, apoptosis, and tumor process. They can provide valuable information about the pathological process and serve as markers of the corresponding diseases.

## MAJOR HEALTH THREAT FROM COSMIC RADIATION

### Injury to the central nervous system

Space flight conditions (SFC) significantly affect the operating activity of astronauts during deep space missions [101–103]. Ionizing radiation, especially GCR creates a risk for the normal functioning of the central nervous system, with acute and chronic exposure leading to alterations in the cognitive abilities, reduction of motor functions and behavioral changes [104]. In contrast to orbital flights, leaving the Earth's magnetic field drastically increases the exposure to ionizing radiation (IR) and, above all, high-energy nuclei component of cosmic rays (HZE). Thus, during a 3-year-long mission to Mars, 13% of neurons in the central nervous system (CNS) will be permeated at least once by an iron ion, while at the same time, ~ 50% of neurons in the hippocampus will be permeated by charged particles with an atomic number greater than 15 [105]. There are a lot of disparate data about the detrimental effects of the SFC onto the cognitive abilities, and on the mechanisms underlying neurodegenerative disorders [106, 107]. To date, the neurochemical and molecular mechanisms underlying the cognitive impairments resulting from the effects of SFC are not clearly understood; even information about the potential risks for the CNS is contradictory [108, 109].

The most harmful component of GCR is the HZE particles, e.g. 56Fe. In rodent models, exposure to even small doses of 56Fe ions, was shown to induce pronounced deficits in hippocampus-dependent learning and memory. In particular, a sharp decrease in spatial memory and orientation in the Morris water maze and Barnes maze were observed after exposure to 56Fe ions in doses 0.1-1 Gy [110] [111] [112] [113] [114]. Current estimates place the relative biological effectiveness (RBE) for 1 GeV/u 56Fe particle-induced hippocampal memory impairment at around 50 [110]. Acute exposures of 48Ti ions in doses 0.02-0.2 Gy (1 Gev/n) significantly reduced the mean spatial memory of the rats at three months after exposure, and significantly increased the percentage of rats with severe impairment, which manifested itself in subpar performance [115]. Indeed, 7, 11 and even 15 months following exposure to 56Fe ions in doses 1-2 Gy, the irradiated groups fared significantly worse on the ascending fixed-ratio operant task (bar pressing for food reward) than the controls.

Rats exposed to proton doses of less than 3 Gy suffered disturbances in conditioned taste aversion 3 days following exposure [116] while doses of 3-4 Gy produced transient direct deficits in open field exploratory behaviour, acoustic startle habituation and rotarod performance in mice [117]. In a rat model, irradiation with high-energy protons in doses of 1-2 Gy induced a decrease in the efficacy of test performance in T-maze. The treatment caused a decrease in the concentration of catecholamines in the prefrontal cortex and 3-MT, a metabolite of dopamine, in striatum [118].

Exposure to 1 Gy of 12C ions drastically reduced the content of 3-MT, 3,4-dihydroxyphenylacetic acid, 5-hydroxyindoleacetic acid and serotonin in the prefrontal cortex of rats [119]. This indicates that the prefrontal cortex is the most sensitive area of the brain in terms of the deleterious effects of HZE on the content of monoamines. Worth mentioning, the changes in the content of neurotransmitters regions rich in dopaminergic neurons, directly affect the emotional and motivational behavior, attention, spatial learning and contextual memory [120] [121] [122]. Finally, HZE can induce the strong disturbances in the CNS, especially in spatial memory and orientation. These disturbances may be critical for performance of astronauts in deep space missions. The non-radiation SFC, especially microgravity, can significantly change the effect of IR injury. Surprisingly, the SFC applied in combination may antagonize each other, for instance, IR eliminated the cognitive deficits occurring in response to the AS. The need for future experiments to further investigate this effect and its mechanisms is evident.

### Cardiovascular diseases

The impact of prolonged exposure to microgravity on cardiovascular system has been well characterized. Tachycardia, baroreflex dysfunction, and reduced physical capacity were observed in experimental animals as soon as in 14 days after microgravity modeling [123]; [124]. Similar changes were observed in humans under conditions of modeled microgravity or after space missions [125]. Long-term sympathovagal imbalance increases the risk of fatal arrhythmias [126] and is the negative risk factor in cardiovascular disturbances and strokes [127] [128]. In astronauts, arrhythmia episodes were recorded during performance under stress [129]. NASA has recently reported increased cardiovascular mortality among lunar astronauts [130]. The authors attribute the observed phenomenon to the effect of lunar radiation on the endothelium. However, a recent report by Cucinotta et al suggests that such conclusions should be revisited due to methodological deficiencies in the analyses by Delp et al. and that circulatory disease due to space radiation does not contribute to mortality in astronauts [131].

### Immune effects

Given that the organs of the immune system, including bone marrow and lymph nodes are some of the most radiation-sensitive tissues in the human body [17], it is of little wonder that abnormalities in the functioning of the immune system have been observed in astronauts as early as the 1970s [132]. In this regard, a depressed immune system in astronauts can potentially increase the incidence of infection, acute and chronic inflammation, and potentially carcinogenesis, while an enhanced immune response is associated with allergies and autoimmune diseases [133] [134]. Therefore, in combination with the greater virulence of pathogens in space [135], immune system dysfunction is of major concern for the expansion of human presence beyond Earth's orbit [136].

The human data on the precise impact of cosmic radiation on the immune system is lacking, mostly due to the fact that there have not been enough manned missions outside of the Earth's magnetic field. *In vitro* investigations show that the d0 (the mean lethal dose) values for various lymphocyte lineages range from 0.25-0.35 Gy [137]; by comparison, an average for mammalian oxygenated cells is around 1.5 Gy. Simulated cosmic radiation experiments performed on lymphocytes also indicate extensive chromosomal exchanges as a result of irradiation, the impact of which can be somewhat mitigated by shielding [138].

Data from animal model experiments performed on Earth is in accord with the above: in a rat experiment, whole-body 1 Gy irradiation produced an acute decrease in the concentration of circulating lymphocytes [139]. Subsequent experiments simulating galactic cosmic irradiation and solar particle events on mice demonstrated both chronic and acute deleterious effects on lymphocyte populations, functioning and chromosomal stability, with circulating B lymphocytes being the most affected, followed by T-cells and natural killer cells (reviewed in [17] and also in [140]).

In the context of radioprotection, previous studies investigated whether pre-exposure to radiation might have an impact on an individual's radiation sensitivity of the immune system. So far, different studies gave contradictory results [141] [142] [143] [144]. Therefore it is worthwhile to further investigate whether pre-exposure to low-dose radiation may offer some degree of radioprotection.

### Cancer and aging

The concept of DNA being the main target of ionizing radiation, combined with the classical views of molecular biology that DNA mutations are the main initiating and propagating events driving cellular neoplastic transformation and carcinogenesis [145], has led to the acceptance of the risk of fatal cancer as the major health risk of exposure to radiation [21]. Predicting such risks and their management is implemented in the current international radiological protection system that is comprised of a number of international and national advisory (e.g. ICRP, UNSCEAR) and regulatory bodies (e.g. NCRP, NIOSH, CNSC). Their cancer risk models and guidelines are predominantly based on the findings of the epidemiological life span study of atomic bomb survivors in Japan [146]. Although the exposure doses in those human cohorts were high, the derived relationship between dose and cancer is extrapolated back to low doses to calculate cancer risks encountered upon environmental or occupational exposure to low doses. This is accomplished with the help of a dose and dose rate effectiveness factor – accounting for sparing of the effect of chronic low-dose exposures – and a radiation quality factor that is based on relative biological effectiveness (RBE) of a particular radiation type and is linked to a LET value. Since high-LET particles – known for their high RBE - are a substantial component of cosmic radiation, fatal cancer risks associated with space missions are an obvious and a serious concern and limits space mission length.

Cancer and aging are intricately related [147]. Indeed, aging, as a factor, is the largest contributor to cancer [147, 148]. A global view of how age affects the host was recently discussed by López-Otin et al. [85] categorizing nine “hallmarks of aging” in the host, but minimal evidence was included towards the direct impact of these hallmarks on cancer progression. Molecular factors involved in the hallmarks of aging are impacted by the individual organs, which are usually not considered a factor in carcinogenesis unless discussing the immediate tumor microenvironment [149]. Changes with the immune system as a function of age stemming from the spleen have potential to affect cancer risk and tumor progression [150]. Limited research exists on cancer risk as a function of age and sex for the interplay between organs. Astronauts typically have an age range of 35-55 years old [151] providing potential biological differences in the host as a function of age that can impact tumor progression. Age and sex are typically studied together obscuring the effects of a central determinant on overall cancer risk. Understanding how systemic host change as a function of age and HZE irradiation will greatly improve cancer risk models for space travel.

In mice, genetic modifications that cause increases in lifespan are also able to concurrently reduce cancer rates [152] and vice versa [153]. Taken together, the model of aging-related accumulation of mutations (Lopez-Otin, 2013) and the multistep mutational theory of carcinogenesis [154] explain well such a strong connection. Although somewhat simplistic and not capable to explain some experimental observations (e.g. leveling off of cancer incidence at advanced age), this explanation may be useful for predicting cancer risks upon exposure to radiation since many observations related to aging can be used to draw connections to cancer. For example, disruption of DNA repair or DNA damage response pathways are frequently observed in both aging and cancer phenotypes [155, 156]. Since epidemiological studies of cancer incidence among astronauts are not practical due to low group size and associated low statistical power, terrestrial studies examining HZE particles-induced changes in various endpoints related to cancer and aging are of primary importance for improving cancer risk models for cosmic radiation exposures.

Modeling cosmic radiation on Earth is extremely difficult due to its very complex particle spectrum and the presence of very high energy nuclei. However, various laboratories built around large nuclear facilities, such as accelerators, have used heavy ions of ^56^Fe, C, He, protons, and some others for studying the effects of HZE particles on a range biological endpoints, such as cancer, systematic response to radiation induced cancer, and DNA damage in both animal and cell culture models [157]. *In vitro* or *ex vivo* studies offer greater opportunities for detailed characterization of RBE as a function of LET for various endpoints related to carcinogenesis, such as chromosomal aberrations, mutations and neoplastic transformation when compared to *in vivo* studies, but are harder to relate the impact on aging. RBE of HZE particles for induction of neoplastic transformation in mouse C3H10T1/2 cells varied between 2 and 10 depending on the LET value of a particular heavy ion [158]. These RBE values may have been underestimated due to the use of X-rays instead of γ-rays as a reference radiation and the use of readouts at 50% cell survival levels. Using chromosomal aberration induction in human peripheral blood lymphocytes exposed *ex vivo* to various heavy ions, RBE values of 10 and 30 were produced for cycling and interphase cells, respectively [159, 160]. Interestingly, these studies independently revealed a similar pattern of RBE dependence on LET by using varying energies of Si nuclei [160] or different nuclei [158], with the maximum RBE observed at around 100 keV/μm. Similar RBE were shown for HPRT mutations [161].

Although very limited in number, animal studies in general show substantially higher cancer rates when compared to low-LET radiation exposures. Thus, HZE irradiation was 20-40 times more effective in inducing Harderian gland tumors in mice compared to low-LET irradiation [39, 162]. This effectiveness was even increased when a single acute dose was split into several fractions, indicating that the attenuation effect seen for low-LET radiation at low dose rate exposures is not present for HZE nuclei [163, 164]. Similar RBE estimates were obtained for skin and mammary tumors in rats exposed to Fe nuclei; however, no changes upon fractionation were found in RBE values [165–167]. Interestingly, Fe particles induced acute myeloid leukemia with the same efficiency as 137Cs γ-rays [162], consistent with α-particle results in mice [168] and the thorotrast patients study [169]. More recently, Miousse et al. [170] showed that epigenetic alterations related to genomic DNA methylation, rather that accumulation of DNA damage, were observed in bone marrow hematopoietic stem cells of mice exposed to 56Fe heavy ions. With respect to aging, irradiation of fish embryos with 56Fe particles, but not γ-rays, lead to increased levels of lipid peroxidation products and other oxidative metabolism pathology, suggesting a higher potential of HZE ions to affect aging when compared to low-LET radiation [171]. Lastly, studies of peripheral blood lymphocytes from human astronauts flown on various length International Space Station missions showed increased levels of chromosome aberrations [172] and their stability over time indicating higher complexity and carcinogenic potential [173]. Overall, *in vivo* results are consistent with *in vitro* studies and confirm substantially higher capacity of the HZE component of cosmic radiation to trigger cancer-related changes compared to low-LET radiation.

In addition, to the factors mentioned above recently miRNAs have been implicated in cancer risk and space radiation. MicroRNAs (small non-coding RNA molecules with a negative and post-transcriptional regulation on gene expression) have recently been implicated in age, sex, radiation, and radiation bystander effects. It is known that each miRNA can target hundreds of mRNAs, which predicts that over half of the existing human transcriptome is regulated by miRNAs [174, 175]. A bystander response to low-LET radiation has been shown to have a sex-specific deregulation on miRNA signature in non-exposed spleens [176]. Evidence also suggests a tissue-specific coordinated pool of miRNAs contribute to the “hallmarks of aging” [177]. These pools of miRNAs are also shown to impact both innate and adaptive immune responses, which can be altered by cytokine stimulation from such factors as TGFβ1 [174, 175, 177]. Recent studies have also started to show the importance of certain miRNA signatures driving cancer progression [178–181]. Studies involving low-LET radiation have demonstrated specific miRNA radiation-dependent signatures [182], but little is known on the potential impact of miRNA and HZE irradiation. Overlap exists between the miRNA signatures in various cancers, age related miRNAs, and miRNAs associated with low-LET radiation, but little is reported on how all these factors uniformly impact cancer progression and effect cancer risk due to space radiation. Further, the impact of where and how the miRNAs affecting these factors (i.e. non-tumor related organs) is not understood.

Although cosmic radiation risks include cataracts, circulatory disease, damage to CNS and others, risks of fatal cancer is the major component and contributor to overall radiation health risks that are being estimated and used for operation and planning of human space missions [183]. Due to the inherent paucity of human data on the space radiation effects on cancer and aging, and difficulties in recreating space radiation fields in the ground-based research studies to better characterize biological effectiveness of such radiation for cancer and aging related endpoints, current health risk estimates carry large uncertainties. It is therefore anticipated that cancer and aging will continue to play a dominant role in future studies to a) minimize the uncertainties to improve health risk management, b) to identify individual factors affecting radiation sensitivity and c) to generate sufficient knowledge for implementing various biotechnological countermeasures described in the next section.

## WAYS TO REDUCE HEALTH RISKS FROM SPACE RADIATION

**Figure 3:**
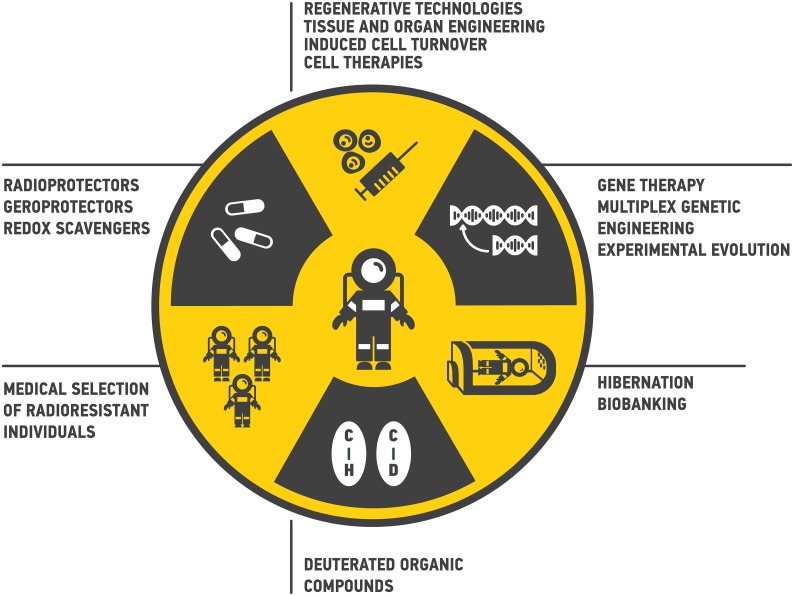
Ways to reduce health risks from space radiation during deep space travels Multiple approaches from medical selection of radioresistant individuals to gene therapy may be proposed.

The principal scheme of the possible interventions to reduce the health risks from space radiation are presented in Figure 3.

### Medical selection of radioresistant people

Countries actively engaged in development of the spaceflight missions, such as United States and Russia, have well-established protocols for selection of the potential candidates. While these selection pipelines differ substantially from one to another [184, 185], *in vitro* adaptive response studies is the only approach widely implemented for the medical selection of the radioresistant individuals [186, 187]. Nevertheless, according to current regulations, the results of such studies are not necessarily taken into consideration along the process of candidates selection for the spaceflight. There is a strong evidence of a wide range of adaptive response among different individuals, suggesting that medical selection of the candidates based on the *in vitro* adaptive response studies is very promising [108, 186, 188, 189]. The wide spectrum of responses may be explained by various rates of DNA damage accumulation and repair [190–194]. The general objective behind such studies is to collect the cell samples from the potential candidates (lymphocytes, skin fibroblasts, etc.) and using these samples for analysis of the adaptive response curves. The implemented readouts may include measurement of cell viability, various indicators of DNA damage and repair markers (H2AX, 53BP1, etc.) after the exposure to IR [151, 191, 195–199]. Moreover, markers specific to the particular DNA repair mechanisms (RAD51, Ku70/80, ATM) may be of a particular interest, as numerous studies have shown higher error-proneness of non-homologous end joining compared to the homologous recombination [29, 30, 36, 200–202]. Application of the comprehensive multi-omics analytical tools on transcriptomic and proteomic data may serve as another useful approach for the individual radioresistance assessment [203–206]. All the approaches discussed in the next chapters, combined with the initial selection of radioresistant individuals, should significantly empower our ability to protect space mission crew members against the HZE-irradiation.

### Small molecule-based interventions

#### Radioprotectors and geroprotectors

**Figure 4:**
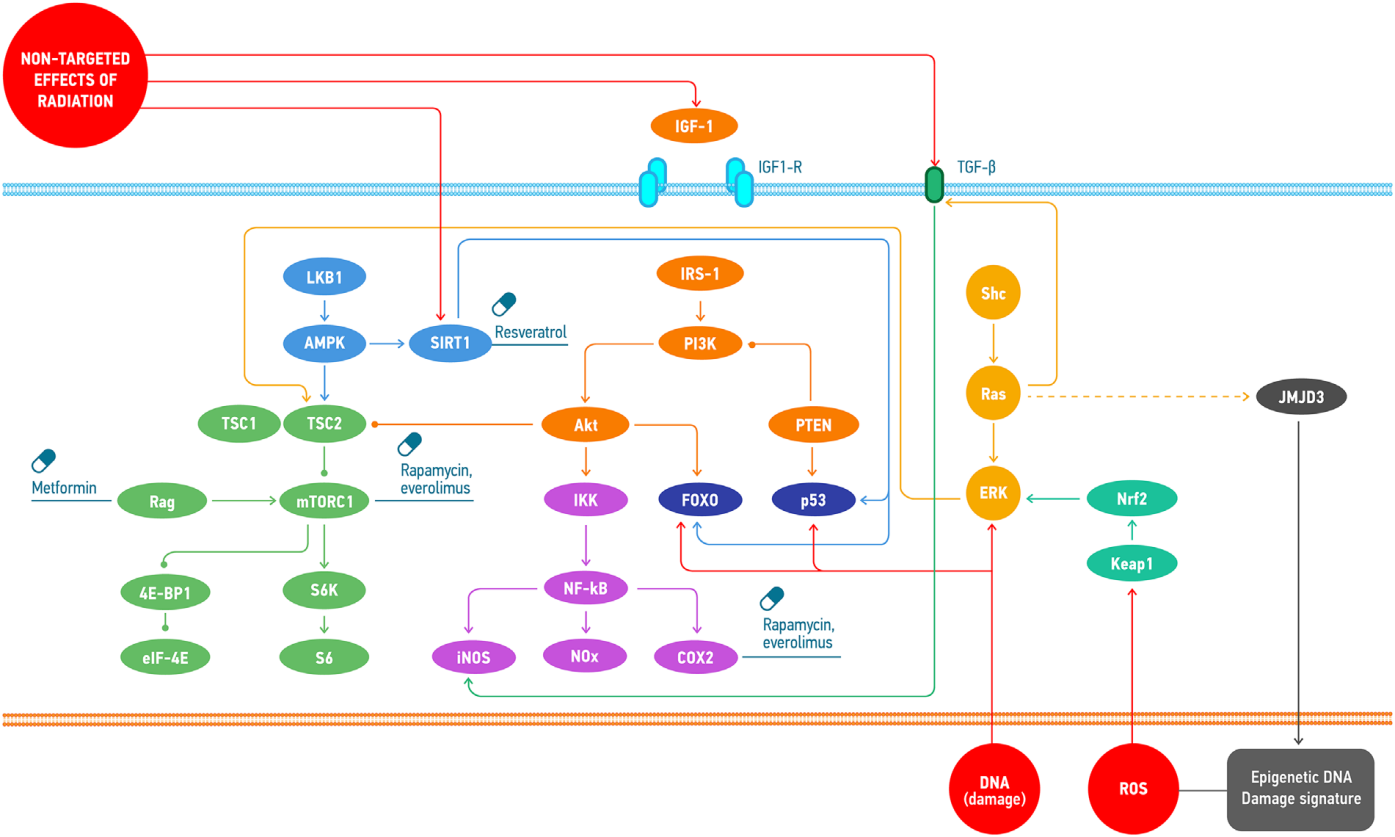
Common molecular mechanisms involved in the response to the effects of space radiation and the geroprotectors affecting the regulation of those Space radiation induces cellular response through the direct DNA damage, ROS accumulation and non-targeted effects. These types of damage provoke distinct signalling mechanisms that may be regulated by the small molecules.

Small molecule interventions for radiation protection have been studied for many years. Such molecules are called radioprotectors. To date, amifostine (Ethyol, MedImmune) is still the only FDA approved radioprotector for treatment of radiation toxicity [207]. Nevertheless, there is still a large potential in this field. Particular pathways playing key role in aging-related pathologies are also involved into ionizing radiation response, for example PI3K/AKT [208], mTOR [209], NF-kB [210], p53 and ATM [211], FOXO, JNK and SIRT1 [212], ERK [213], JMJD3 [214], TGFb [215]. In other words, ionizing radiation causes accelerated aging both at the cell level (stress-induced premature senescence) and in the body [66, 216]. Therefore, it is not surprising that some geroprotectors (pharmacological agents that decrease the rate of aging and extend the lifespan) are able to reduce the damaging effect of ionizing radiation, for instance, curcumin [217], quercetin, (−)-epicatechin, and ibuprofen [218]. At the present day, according to Geroprotectors.org (http://geroprotectors.org/) [219] and DrugAge (http://genomics.senescence.info/drugs/) [220] databases, there are more than 400 potential geroprotectors, compounds that prolong model organism's life span at least in one concentration [221] (Figure 4). The drug repurposing approach may be very useful in search for the new radio- and geroprotectors [222].

#### The role of redox scavengers in radioprotection

Glutathione (GSH) is a tri-peptide redox scavenger abundant in eukaryotic cells. Its capacity to prevent oxidative damage rests in the presence of a cysteine residue in both it and its cognate disulfide (GSSG). More specifically, the thiol group readily participates in radical chemistry, enabling it to nullify the deleterious effects of ROS. Indeed, *in vitro* assays show that GSH at concentrations of 100 μM (with the physiological concentrations being in the mM range) was capable of protecting equimolar nucleic acids from γ radiation-induced degradation, even at doses reaching 400 Gy [223]. On a cellular level, it has been known for a long time that exogenous supply of glutathione can serve as a radioprotectant – lymphoid cell lines subjected to GSH depletion exhibit radiosensitivity to 7 Gy of γ radiation, which is rescued by GSH repletion [224]. In addition, GSH can serve as a useful biomarker of cellular oxidative stress and aging; in a rat model, young specimens had significantly higher levels of intracellular GSH, which correlated negatively with the extent of DNA damage [225]. There is evidence that the radioprotective properties of glutathione can be exploited therapeutically. For example, diselenonicotinamide (DSNA) is a synthetic organometallic compound, the administration of which in a cellular context protected Chinese Hamster Ovary and murine splenic lymphocyte cells from radiation-induced cell death at doses of 25 μM administered before and even after the irradiation of 1-12 Gy [226]. Another compound, silymarin, when administered orally for 3 days in a mouse model resulted in a 67% survival rate after 30 days following lethal 9 Gy γ irradiation, with a concomitant increase in the activity of enzymes participating in the GSH pathway [227].

The coenzyme nicotinamide adenine dinucleotide (NAD) and its cognate reduced form, NADH, is a reducing equivalents carrier known for participating in various metabolic reactions paramount to cellular respiration. Whilst originally thought of mostly as a metabolic ancillary, evidence has emerged of it being an important indicator of the redox state of the cell, and concomitantly, a factor in aging and tissue regeneration. Early reports at the turn of the decade suggested that NAD levels are negatively impacted by high-fat diet and linked to type 2 diabetes (T2D), with NMN (a precursor to NAD) supplementation being capable of improving insulin sensitivity and glucose tolerance in a mouse model of T2D [228]. Strikingly, NAD levels were shown to decrease with age, and along with them the activation of a histone deacetylase family of proteins, known as Sirtuins (SIRT1-7). Sirtuins, responsible for the regulation of gene silencing, use NAD as a cofactor and also happen to be involved in aging and disease. For instance, one of the targets of SIRT2 is a mitotic checkpoint kinase BubR1, the levels of which have been associated with longevity. In a mouse model, the age-related decline in BubR1 levels was predicated upon the decline in NAD levels and vice versa: overexpression of SIRT2 or administration of NMN increased BubR1 levels [229]. Furthermore, it was later shown that NMN was capable of ameliorating some of the more deleterious effects of aging in a mouse model, with a 12-month-long trial demonstrating NMN's positive effects on metabolism and physical fitness, as well as vision [230]. The recent work from the Sinclair lab at Harvard Medical School further establishes the link between NAD and aging by supplementing it with their research on DNA damage. PARP1, a protein heavily involved in ssDNA damage repair, also uses NAD as a cofactor. Interestingly, PARP1 gets sequestered and inactivated by a protein called DBC1, which incidentally also happens to inactivate SIRT1. This led to the hypothesis that elevating NAD levels by administering NMN will lead to a decrease in PARP1 inactivation by DBC1, leading to an increase in the rate of DNA repair. A number of observations confirmed this, most notably the fact that older mice tended to have lower hepatic NAD concentrations and higher amounts of the PARP1-DBC1 complex, both of which were improved by NMN [231, 232]. The key region in DBC1 responsible for PARP1 sequestration appears to the Nudix Homology Domain (NHD), which is present in a large number of proteins involved in DNA repair [233]. Thus, NAD and its precursor NMN appear to be key regulators of protein-protein interactions and DNA damage response networks, and as such NMN supplementation needs to be taken seriously as a possible branch of radioresistance.

#### Hypostasis, hypothermia and biobanking

Soon after the launch of the first human space missions, the lead designer of the Soviet space program, Sergei Korolev, has began to develop an ambitious project of a manned flight to Mars. His idea was to put the crew into a hibernation state during lengthy space travel. Although a member of the Soviet Academy of Sciences, Vasiliy Parin, has termed this hypothetical process as ‘hypobiosis’ [234], the more currently used term, ‘hypostasis’, which more accurately reflects this physiological process, since the desired hibernation state requires not only a decrease of the metabolic rate, as we will describe later, but also the slowdown of all vital bodily processes down to a complete standstill (stasis), with the possibility of subsequent recovery [235]. The development of a hypostatic state (HS) is based on the repression of complexes III and IV of the mitochondrial respiratory chain and transition of the warm-blooded organism into the bioenergy of cold-blooded animals (i.e. bioenergetics of glycolysis and the Krebs cycle). The extensive experimental work on the laboratory animals which attempted to generate the HS at different temperatures, various durations and degrees of depth, has led to surprising discovery that mammals have increased resistance to the influence of extreme factors. Among others, such factors include the deeper levels of hypothermia to 0°C, deadly doses of radiation, a state of severe irreversible shock blood loss, hypobaric conditions of oxygen deficiency (18 km) and deadly magnitude of the overload (up to 50 g) [16]. Although the hypostatic state has a potential to greatly reduce, if not fully eliminate, the radiation-associated risks, and may have a wide range of applications for future deep space missions, at the present time, it is quite far from the practical usage and requires years of extensive research before it can be applied in humans.

On a more futuristic note, departure of humanity from Earth to colonize other objects/planets, may not be compatible, even with all the biomedical and biotechnological advancements of the time, with the long-term survival of the human population due to radiation damage encountered during the spaceflight itself. In such case, transportation of a bank of cryopreserved germ cells destined to give rise to a new population of humans on another planet/object seems to be a reasonable alternative. Indeed protecting a bank of frozen cells from cosmic radiation physically is obviously more feasible than protecting a population of humans. Yet, none of the humans presently living on Earth may witness such endeavour of our civilization and there is hope that a variety of other strategies described here that could make human sufficiently radioresistant to withstand multi-year exposure to cosmic radiation will eventually succeed [236].

### Gene therapy for deep space exploration

#### Overexpression of endogenous and exogenous antioxidants

One promising strategy is to overexpress endogenous antioxidants via administration and expression of their cognate transgenes [237]. A variety of teams have reported increased radioresistance following the delivery of magnesium superoxide dismutase (MnSOD) transgenes in a variety of tissues. MnSOD transgenes have been delivered both systemically [238] and in a tissue-specific manner [239, 240] using plasmid/liposomal vectors, resulting in a substantial reduction in early and late-stage irradiation damage.

Note that antioxidants can only protect against reactive oxygen species (ROS) primarily produced by water radiolysis during exposure to ionizing radiation. As such, a fraction of the DNA damage induced by direction ionization of DNA cannot be prevented by antioxidants. The majority of DSB and SSB generated by low-LET are induced by ROS (“indirect effect”) and thus antioxidants would be most efficient against low-LET. In contrast, the majority of damage for high-LET are due to direct ionization of DNA making antioxidants less efficient.

However, constitutive overexpression of endogenous antioxidants could detrimentally interfere with redox signalling [241]. Additionally, while current clinical evidence suggests that dietary antioxidant supplementation does not interfere with radiation therapy for cancer [241, 242], there is a minimal possibility that constitutive overexpression of antioxidants could reduce the clinical efficacy of radiation therapy for cancer in the event of subsequent carcinogenesis. This may happen due to enhanced DNA repair capabilities in cancer cells. Constitutive expression would also presumably provide anticarcinogenic benefits though prior to carcinogenesis due to constant reduction of endogenous ROS. It would therefore be prudent to use inducible expression constructs (e.g. inducible promoters), so that if carcinogenesis were to occur the expression of such endogenous antioxidants could be halted by no longer administering the trigger stimulus used by the inducible expression system.

Indeed, an even more robust approach would be use distinct inducible expression systems, such as the use of distinct inducible promoters, on a tissue and organ-specific basis. If the same inducible promoter were used to control the transcription of endogenous antioxidants in all tissues and organs of the body then halting the administration of that inducible promoter's trigger stimulus in the event of carcinogenesis would halt the radioprotective benefits conferred by antioxidant overexpression in all tissues and organs of the body. If, however, distinct inducible promoters were used to control antioxidant transcription and expression in distinct tissues and organs then halting the administration of an inducible promoter's trigger stimulus could be restricted to the tumor's tissue of origin without negating the radioprotective benefits of antioxidant overexpression in all other tissues and organs using a different inducible promoter.

#### Overexpression of endogenous and exogenous DNA repair genes

Overexpression of human and non-human DNA repair proteins in mammalian cells has been previously explored as a strategy to enhance the efficiency of endogenous DNA repair and to reduce mutagenesis and associated carcinogenesis in animal models [243, 244]. This constitutes an intriguing strategy that could be utilized to enhance radioresistance of humans for the purposes of deep space exploration. Some instances (such as overexpression of O6-alkylguanine DNA alkyltransferase and yeast AP endonuclease) have led to enhanced protection from endogenous and exogenous mutagens, while others (e.g. overexpression of alkyl N-purine glycosylase and DNA polymerase β) resulted in increased genome instability [243, 244]. Continued progress in elucidating which DNA repair proteins result in increased DNA repair capabilities and which do not will aid in the eventual determination of which DNA repair proteins can be usefully overexpressed for the purposes of enhancing radioresistance in humans.

However, as with the case of overexpression of endogenous antioxidants, even those DNA repair proteins that show largely beneficial effects in terms of mutagenesis protection may reduce the clinical efficacy of radiation therapy for cancer in the event of subsequent carcinogenesis due to enhanced DNA repair capabilities in cancer cells when constitutively expressed [245], while simultaneously enhancing protection from carcinogenesis prior to actual malignant transformation. It would therefore also be prudent to use inducible expression constructs (e.g. inducible promoters) in the case of overexpression of DNA repair genes as well, and to explore the possibility of using tissue and organ-specific inducible promoters to open up the possibility of halting the administration of the inducible promoter's trigger stimulus exclusively in the tumor's tissue of origin, while still maintaining the radioprotective benefits of DNA repair gene overexpression in all other tissues and organs.

DNA repair processes have evolved to repair isolated endogenous DNA damage. Therefore, cells can be overwhelmed with the very unlikely event of multiple DSB occurring at the same time in close proximity within the nucleus. It has been shown that the probability of genomic rearrangements resulting from incorrect end joining is higher [246–248]. Movement of individual DSB into common repair domains makes such genomic rearrangements more likely than previously anticipated [35] and such phenomenon has been coined “DSB clustering”. Computer models have shown that spatiotemporal distribution of DSB is in fact sufficient to predict individual cell death probability [249]. In addition, DSB clustering is a lot more probable for HZE due to the significant proximity of DSBs along particle tracks (see Figure 2). As such, one can predict the LET dependence of RBE for clonogenic survival by simply taking into account the spatiotemporal distribution of DSB and the inherent clustering properties of DSB [36, 249–251]. In this model, two isolated DSB are less toxic to a cell than two clustered DSB and thus shows that DNA repair is not as efficient in dealing with HZE-induced DSB. In addition, DSB induces complex DSB [252], as discussed in previous section, which are poorly repaired by nonhomologous end joining (NHEJ), the primary DNA repair mechanism of cell [253]. Therefore, DNA repair gene overexpression will be less efficient for exposure to HZE compared to low-LET.

As previously discussed in detail [36], chromatin density mediates the response to DNA damage. The full mechanism by which this happens remains unclear, but local chromatin structure appears to play a role. Chromatin decondensation around the DSB is believed to be an important trigger for ATM dimer dissociation and subsequent ATM autophosphorylation and activation [250, 251]. We have hypothesized that DSB clustering is also modulated by chromatin remodeling and may differ depending on the cell cycle [254]. Therefore, the ability to modulate the chromatin density is another potential direction to infer protection to the cell, with tightly packed DNA being better protected and thus more resistant to ionizing radiation [255]. Interestingly, high chromatin condensation is typically associated with gene silencing and lower cell activity, just like hypostatic state leads to a full stop in cell activity and radioresistance.

#### Expression of endogenous and exogenous radioprotective transgenes

Another potentially more promising strategy involves the delivery and expression of exogenous and translational radioprotective transgenes. Many organisms (e.g. tardigrades, *Deinococcus radiodurans*) possess remarkable degrees of radioresistance, and if the genes and molecular mechanisms conferring such high degrees of radioresistance can be elucidated and translated to humans via gene therapy then this would constitute a much more effective strategy to enhancing radioresistance in humans for the purposes of deep space exploration than the overexpression of endogenous radioprotective genes, which are comparatively limited in their radioprotective effect. Tardigrades, for instance, are not only remarkably resistance to the damaging effects of irradiation [14, 256]; but much more remarkably have been shown to survive direct exposure to space in low Earth orbit [257].

A nuclear protein thought to aid in protection of DNA from the damaging effects of ionizing radiation in tardigrades, termed Damage suppressor (Dsup), has recently been discovered [258]. This protein co-localizes with tardigrade DNA, and has also been shown to co-localize with nuclear DNA in human cultured HEK293T cells. The protein is highly basic, which suggests that it may associate with nuclear DNA via electrostatic interactions, although the protein's mode of interaction with DNA remains to be comprehensively elucidated. To examine the possibility that the association of Dsup proteins with nuclear DNA aids in protecting DNA from radiation damage, the team stably expressed Dsup using the constitutive CAG promoter, and confirmed co-localization with nuclear DNA via immunocytochemistry. When the HEK293 cells were exposed to 10 Gy of X-ray irradiation they found that the transfected cells had roughly half as many SSBs than the control group. They also examined its effect on the amount of DSBs in the transfected cells via a neutral comet assay and via analysis of the number of γ-H2AX foci (an indicator of DSBs), and using both assays found a 40% reduction in DNA fragmentation in the transfected cells compared to the control group. They also analyzed cell viability following irradiation, as human cells often lose proliferative ability following 3-7 Gy of X-ray irradiation, and found that following 4 Gy X-ray irradiation the transfected cells had slightly increased viability, a much more normal morphology and higher proliferative ability than the irradiated control cells. Thus, the delivery and expression of Dsup transgenes *in vivo* represents a promising candidate for potential exogenous radioprotective transgenes that would aid in establishing enhanced radioresistance in humans for the purpose of deep space exploration.

#### Characterization of the genetic determinants of high human radioresistance in conjunction with clinical translation via multiplex genetic engineering

Single-nucleotide polymorphisms (SNPs) are variations in single nucleotides that occur at specific positions within an organism's genome and that are present in an appreciable portion of a population (e.g. > 1%). As known from radiation oncology, human individuals differ in acute radiosensitivity to high therapeutic doses of radiation [259]. This is driven by genetic germline variation, 90% of which is expressed as SNPs [260]. Limited and inconclusive evidence also exists that suggests a link between genetic variance and health risks associated with low-dose radiation exposures [261] and this issue has been identified as one of the most important questions in low-dose radiobiology (EU low-dose research program DOREMI, http://cordis.europa.eu/result/rcn/183770_en.html). The SNP profile of a given individual characterizes the uniqueness of their genome. As such, individual differences in radiosensitivity within the human population can be characterized by differences in individuals’ SNP profiles and epigenetic profiles. This opens the door to one conceivable strategy – known as genome-wide association studies or GWAS - for determining the SNP and epigenetic profiles of individuals highly radioresistant to low-dose radiation: performing life-long studies of radiosensitivity in very large human cohorts with known radiation exposure doses. These exposures could be both environmental in areas with high natural background radiation levels [262], and occupational, such as InWorks cohorts [263]. One could conceivably find populations that have naturally been exposed to low-dose radiation, such as airline pilots and crews, populations exposed to background radon or on top of granite, etc. In this way the genomic and epigenetic profiles of individuals highly resistant to low-dose radiation could be characterized via RNA SEQ and genome-wide epigenetic profiling of each individual in the cohort. The incidence of cancer (minus the baseline natural rate of cancer incidence) and other diseases in which irradiation is causally implicated would be tracked and associated with individuals’ SNP profiles. In this way, the specific SNP profiles of highly radioresistant portions of the cohort (i.e. those with the lowest incidence of cancer) could be identified, and those SNP variants that are shared among the most radioresistant portions of the cohort identified via statistical analysis. The result of such a large-scale study would be the most likely set of candidate SNP variants that confer high radioresistance in the human population. This strategy resulted in significant advances in understanding a role of genetic variability in several diseases and identification of molecular targets translatable into clinics [264]. Particularly encouraging is the fact that successes have been achieved for complex phenotypes – cancer being also a complex multistep disease - that are highly polygenic, such as psychiatric symptoms and autoimmune diseases [264].

Although the cost of SNP profiling has dropped significantly over the past few years and it is conceivable to carry out such an SNP study in a large cohort, low incidence of cancer due to low-dose radiation exposures may limit if not preclude the ability to conduct such large-scale SNP studies. Next generation sequencing (NGS) technologies may help counter this issue, but at the cost of substantially increasing the cost of such studies. Additionally, the use NGS as opposed to SNP profiling may help identify those rare genetic variants that have a role in radiosensitivity but not detectable by SNP profiling. Gene-wide association studies (GWAS) would provide the best possible association between natural genetic variation and radioresistance, and the best available means of characterizing the genetic and epigenetic determinants of radioresistance and radiosensitivity in the human population.

If the specific SNP variants conferring increased radioresistance in the human population can be characterized, this opens up the possibility of using targeted gene editing technologies (e.g. CRISPR/Cas9) to replicate those SNP variants in individual humans. While the scale involved is clinically unprecedented, likely requiring hundreds to thousands of nucleotide modifications per genome, and hundreds to thousands of modifications per genome per cell unless germline genetic engineering were employed, it nonetheless constitutes a foreseeable future strategy for enhancing radioresistance in humans for the purposes of deep-space exploration.

#### Generation and characterization of enhanced radioresistance via experimental evolution in conjunction with clinical translation via multiplex genetic engineering

An additional strategy is to employ experimental evolution [265] so as to generate populations of model organisms or human cells with enhanced radioresistance by exposing each generation to high levels of radiation and allowing the survivors to breed, followed by the characterization of the genetic determinants of the resulting enhanced radioresistance phenotype and the subsequent clinical translation of such genetic determinants in humans via multiplex genetic engineering. As a case study, a team led by Michael Cox produced a highly radioresistant population of *Escherichia coli* (i.e. 3-4 orders of magnitude more radioresistant to 3000 Gy of IR than the originating generation). Of the 69 mutations generated, only 3 nucleotide changes in the DNA metabolism genes *recA*, *dnaB*, and *yfjK* accounted for the vast majority of the extremophile phenotype, with 4 other mutations providing small but measurable contributions to the phenotype [266]. By employing this method using model organisms with DNA repair pathways conserved in humans, populations with extreme radioresistant phenotypes can be generated and the genetic determinants underlying the extremophile phenotype could be characterized and translated to humans using multiplex genetic engineering (e.g. via the CRISPR/Cas9 system) in a manner similar to that proposed for translating natural highly-radioresistant SNP profiles in the human population to individual human patients.

### Apoptotic & regenerative technologies

The above strategies involve enhancing radioresistance in humans. An alternative strategy involves employing therapeutic modalities in regenerative medicine [267–269] to facilitate the elimination and substitution of endogenous cells damaged by cosmic irradiation. The present strategy can be subdivided into two distinct approaches consisting of random elimination and substitution on the one hand and targeted and radiation-responsive elimination and substitution on the other.

Random elimination and substitution would involve ablating and replacing biological systems in a random fashion, independent of the level of irradiation damage they have acquired, in order to eliminate and replace any cells, tissues and organs that may have sustained enough irradiation damage to put them at substantial risk for carcinogenesis. The scale of elimination and replacement could take place on the level of individual cells, tissue or organs depending upon the specific modality of regenerative medicine employed. For instance, whole tissues and organs could be eliminated and replaced via the use of engineered tissues and organs, periodically according to the calculated rate of damage and consequent physiological detriment (i.e. by measuring or estimating the amount of cosmic radiation exposure and replacing a given tissue or organ once within the period of time it is calculated to have experienced enough cosmic radiation exposure to result in serious physiological detriment, e.g. a high risk of carcinogenesis).

Alternatively, at the level of cells, a recently proposed therapeutic modality in regenerative medicine titled Induced Cell Turnover (ICT) could be employed to periodically abate the endogenous cells constituting an individual's tissues and organs and replace them with either patent-specific or HLA-matched human pluripotent cells (hPSCs) in a gradual and multi-phasic manner [270, 271]. The proposed procedure consists of the quantitative and qualitative coordination of targeted endogenous cell ablation with hPSC-derived exogenous cell administration to mediate partial and whole-tissue/whole-organ replacement at the cellular level, mediated in a gradual, multi-phasic manner so as to minimize the spatiotemporal distribution of ablation and replacement for the purpose of maintaining the homeostatic (i.e. structural and functional) integrity of tissues and organs throughout the course of the procedure. It is distinct from normative cell therapies by the quantitative and qualitative coordination of endogenous cell ablation with exogenous cell administration for the purpose of creating readily-accessible vacant niches for administered cells to engraft and is presumed to increase the engraftment efficacy of administered cells.

While the authors originally proposed the therapeutic modality as a means of periodically attenuating the accumulation of age-related phenotypic deviation for the purposes of treating age-related disease, it could potentially be employed for the purposes of periodically negating acquired irradiation damage in the tissues and organs of humans undergoing long-term space travel, along timescales and employing ICT rates and spatiotemporal distributions that are calculated to facilitate the induced turnover (i.e. elimination and replacement) of cells at the rate at which they are expected to acquire enough irradiation damage to put them at substantial risk of malignant transformation [271]. The ultimate aim, then, would be to facilitate the induced turnover of tissues and organs at least once within the span of time it would take to put them at high risk for carcinogenesis as a result of acquired cosmic irradiation damage, which is a function of the duration and level of exposure to cosmic radiation that spacefaring humans undergo. Indeed, such a proposal could have positive potential implications as a preventative strategy for carcinogenesis in general, regardless of exposure to cosmic irradiation. The above strategy could also potentially be used to attenuate the negative physiological effects of long-term exposure to microgravity when specifically targeted to the constitutive and supportive cells of muscular and osseous tissues.

**Figure 5:**
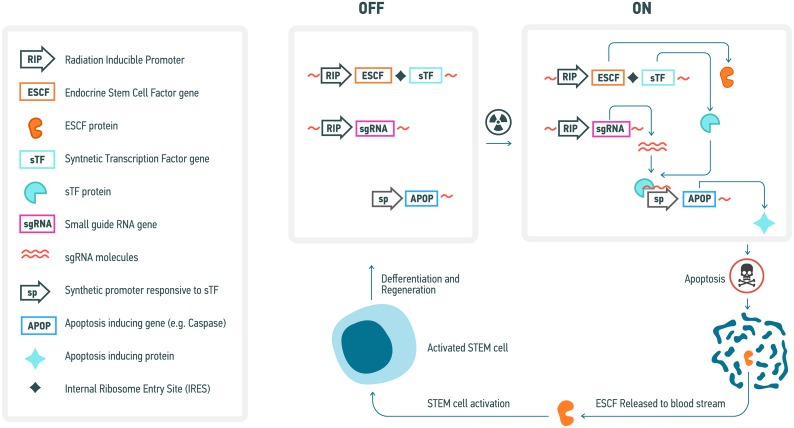
Conceptual diagram of a genetic system for elimination of radiation-damaged cells and subsequent inducible stem cell activation and regeneration of affected tissues A variety of specific genetic elements that could be used in such system have previously been described (see text for further detail).

The second approach, involving the targeted and radiation-responsive elimination and substitution of cells, consists of engineering the cells constituting an individual's tissues and organs to undergo apoptosis in response to the acquisition of irradiation damage in a targeted fashion, in accordance with either the self-detected levels of cosmic irradiation they are exposed to or the self-detected amount of irradiation damage that they acquire, such that they commit apoptosis at levels of acquired irradiation damage (e.g. clustered DNA damage) below the levels normatively required to induce the endogenous apoptotic response to DNA damage. This would most likely consist of the use of a radiation-responsive promoter that drives expression of a synthetic or a recombinant transcription factor that binds very specifically to a synthetic promoter that up-regulates a pro-apoptotic factor (Figure 5). Such system needs to be tuned up such that even slight up-regulation of the synthetic transcription factor would lead to a robust expression of a pro-apoptotic factor, ensuring elimination of a cell damaged by cosmic radiation. Also, this system would need to include a trigger for the activation of somatic stem cells (SCSs) in the case of renewing tissues and engineered hPSC niches in the case of non-renewing tissues so as to replace those endogenous cells that are terminated in response to cosmic radiation exposure. For instance, the radiation responsive promoter could also control the transcription of an endocrine factor, activates and/or upregulates SCS or hPSC endogenous and/or synthetic or translational factors controlling SCS and hPSC proliferation and mobilization in a targeted manner (such that each distinct tissue-specific SCS and hPSC population is equipped with a specific promoter correlating with a distinct synthetic or translational endocrine agent). Obviously the technical realization of such a genetic regulatory system will require substantial effort. However, many elements of it can be suggested using present knowledge. For example, radiation-inducible promoters have been proposed and optimized for use in combination with radiotherapy for cancer and may include the egr-1 promoter or its elements [272], a fusion of dCas9 with multiple copies of the herpes virus transcriptional activation domain VP16 guided to the synthetic promoter of a pro-apoptotic factor by small guide RNA [273], also driven by the radiation-inducible promoter, to ensure its specificity and a lack of activation at the basal condition (Figure 5).

These considerations, however, need to take account of quantitative assessment of the rate of lethal/oncogenic cell damage occurring as a result of exposure to cosmic radiation. Thus, it was estimated that every cell of a human body is traversed by a proton every three days, by a helium ion every month and by a HZE particle every 100 years [274]. Whereas traversal by a proton may not be lethal or mutagenic since only at the end of a track a proton would produce a highly compact ionization track, heavier particles would definitely produce sufficient damage to either kill or produce chromosome damage upon every hit. Therefore, it would be ideal if the trigger of cell removal would sense the outcome of cell response to a particle hit, rather than a hit itself, so that every cell is given an opportunity to successfully repair damage. This would lead to removal of only those cells that failed to properly repair their genomes, helping to maintain the regenerative potential of the body or exogenous sources for longer periods of time.

One potential limitation of such a system would be leakage as a result of its non-linear regulation, and as such stringent preliminary studies and trials to as to optimize its design for liminal leakage would be required. Such an idea is nonetheless presently conceivable and as such constitutes a future research strategy that should be explored for the purposes of minimizing the effects of cosmic irradiation for the purposes of long-term human space travel, and could have additional use as a general preventative strategy against carcinogenesis via the use of both radiation-responsive promoters and carcinogen-responsive promoters.

### Deuterated organic compounds

All organic compounds contain carbon-hydrogen (C-H) bonds. It is, however, possible to synthesize organic compounds that have hydrogen atoms replaced by deuterium (the stable isotope of hydrogen) at specific locations. Due to deuterium's comparatively greater mass with respect to hydrogen, carbon-deuterium bonds require more energy to break and take longer to break than C-H bonds. Consequently, the energy required to break the hydrogen bonds between DNA bases would be greater, and deuterated DNA would be less subject SSBs and DSBs than non-deuterated DNA.

The extent with which we could deuterate a given organism, however, is limited by several potential problems. Specifically, the rate at which bonds between deuterium and other atoms break is lesser than the rate at which bonds between hydrogen and other atoms break due to the kinetic isotope effect (a mechanistic phenomenon where isotopically substituted molecules react at differing rates). Isotopic substitution affects only mass-dependent properties (e.g. vibrational frequencies), but has no effect on the values of atoms’ electronic states or the potential energy surface of a given reaction. Due to the greater mass of deuterium with respect to hydrogen, breaking the bonds holding various deuterated organic compounds together would require more energy, which is a potential problem because organisms have evolved in an environment that requires only enough energy allocation to break bonds between hydrogen and other atoms. In other words, organisms are not energetically adapted to catabolize organic compounds containing deuterium.

A more pertinent potential problem, however, is the fact that the greater mass of deuterium with respect to hydrogen implies that the rate of metabolic reactions may be slower due to the decreased rate of bond-breaking in deuterated organic compounds, which is problematic due to the precise coordination and timing of metabolic reaction rates that organisms are adapted to and that are required to facilitate normative metabolism. It may thus be possible to deuterate an organism by a certain amount without witnessing significantly detrimental physiological effects, but the specific safety threshold would have to be determined through a variety of preclinical studies and clinical trials.

Moreover, incorporation of isotope-reinforced food and water has emerged as a novel means of promoting longevity and the maintenance of several aspects of health in eukaryotes [275]. Several teams have reported that the administration of deuterated water increases the lifespan of yeast in a dose-dependent manner by as much as 80% [275]. In multicellular eukaryotes, however, bodily incorporation of deuterated water above 20% begins to have toxic effects. Nonetheless, incorporation of deuterated water has been shown to either increase longevity or healthspan in yeast, *Caenorhabditis elegans*, *Drosophila melanogaster*, rodents and humans [276–280], and has been found to have anti-cancer effects in rodents [280–282] and humans [283]. Such effects are thought to be the result of protection against DNA damage and mutation from ROS and exogenous mutagens.

An intriguing alternative involves supplying organisms with food containing stable isotopes of carbon, e.g. ^13^C, [281, 282] which has a greater mass than ^12^C (which comprises the majority of natural carbon in organisms), but only ~8% greater than ^12^C, whereas deuterium has a mass 100% greater than hydrogen. This means that while the rate of bond breaking in the C-H bonds of organic compounds having ^12^C replaced with ^13^C would be slower, it may not be so slow as to disrupt the timing and coordination of metabolic reactions or to significantly affect the necessary energy allocation to catabolic reactions required for their ongoing maintenance. However, this would only be capable of potentially reducing the damaging effect of ionizing radiation for organic compounds like proteins, and not DNA because the hydrogen bonds occurring between DNA bases are N-H and O-H bonds rather than C-H bonds, and the problem of irradiation-induced DNA SSBs and DSBs in particular are much more detrimental than irradiation-induced damage to gene products. Furthermore, it is currently extremely costly to produce. It may, nonetheless, constitute an ancillary strategy for reducing the overall damaging effects of radiation to humans as it pertains to deep space exploration, provided that a less costly means of producing it could be developed.

## UTILIZING THE ADVANCES IN ARTIFICIAL INTELLIGENCE FOR DIAGNOSIS AND TREATMENT OF RADIATION- AND AGING-INDUCED DAMAGE

In recent years much progress has been made in the applications of artificial intelligence and specifically deep learning to biomarker development and drug discovery [284]. Deep neural networks (DNNs) were applied to profiling of the biological samples [285] predict the age of a patient using the basic clinical blood tests and identify the most important features [286]. Similar concepts can be applied to other data types including transcriptomic, proteomic, imaging, photographic, activity and physiological data to evaluate the minute changes transpiring during aging or due to the irradiation in space. These minute changes can be addressed with the many interventions described in section VI. A prototype of such a system for monitoring a variety of data types over time called Young. AI was recently launched for testing (www.young.ai). Developing comprehensive predictors of age and accumulated damage using multiple data types on the population level and re-training them on the individual spacefarers is one of the approaches to build the artificially-intelligent health monitoring systems and assess the types of damage and the resulting changes that are difficult to predict with the amount of information available before the long-term exposure to radiation and a variety of other stress factors in space. Space colonists should also have the ability to engage in AI-enabled personalized drug discovery and rapid validation techniques. On Earth the drug discovery and development for a known indication takes many years and the likelihood of approval (LOA) of a drug from phase 1 to the Food and Drug Administration (FDA) approval is 10.4% for all indications [287]. In space this process must be expedited and artificial intelligence can be employed to identify the effective molecules from the existing chemical space [288] or even generate novel molecular structures with the desired set of parameters [289].

## CONCLUSIONS

We have outlined several alternative and complementary strategies for enhancing human radioresistance for the purposes of deep space exploration and colonization using existing biomedical and biotechnological tools and modalities in an attempt to lay the foundation for a comprehensive roadmap towards highly radioresistant humans. While many of the strategies proposed above may seem speculative, they should be considered as a foundation for future research directions. Meanwhile, we have highlighted the link between radioresistance and aging, and have endeavored to show how many of the biomedical and biotechnological modalities discussed in the present review could combinatorially be applied to both enhancing human radioresistance on the one hand and healthy longevity on the other. We also highlighted the need to converge and accelerate the research in radiobiology, biogerontology and AI to enable spacefarers to address the healthcare challenges we may not yet be aware of. Furthermore, given the massive amount of funding allocated to research into facilitating and optimizing space exploration and optimization, we hope to have shown how research into enhancing radioresistance for space exploration could galvanize progress in human healthspan extension, an area of research that is still massively underfunded despite its potential to prevent the massive economic burden posed by the future healthcare costs associated with demographic aging.
